# An ascomycete H4 variant with an unknown function

**DOI:** 10.1098/rsos.231705

**Published:** 2024-02-21

**Authors:** Michel Flipphi, María Laura Harispe, Zsuzsanna Hamari, Sándor Kocsubé, Claudio Scazzocchio, Ana Ramón

**Affiliations:** ^1^ Department of Biochemical Engineering, Faculty of Science and Technology, University of Debrecen, Debrecen, Hungary; ^2^ Instituto de Profesores Artigas, Consejo de Formación en Educación (CFE, ANEP), Uruguay; ^3^ Faculty of Science and Informatics, Department of Microbiology, University of Szeged, Szeged, Hungary; ^4^ Department of Life Sciences, Imperial College London, London, UK; ^5^ CEA, CNRS, Institute for Integrative Biology of the Cell (I2BC), Université Paris-Saclay, Gif-sur-Yvette 91198, France; ^6^ Dpto. de Biología Celular y Molecular, Facultad de Ciencias, Sección Bioquímica, UdelaR, Uruguay

**Keywords:** histone H4 variant, chromatin, ascomycetes

## Abstract

Histone variants leading to altered nucleosome structure, dynamics and DNA accessibility occur frequently, albeit rarely for H4. We carried out a comprehensive *in silico* scrutiny of fungal genomes, which revealed the presence of a novel H4 variant (H4E) in the ascomycetes, throughout the Pezizomycotina, in basal species of the Taphrinomycotina and also in the Glomeromycota. The coding cognate genes show a specific intron/exon organization, different from H4 canonical genes. H4Es diverge from canonical H4s mainly in the N- and C-terminal extensions, showing marked differences in the distribution and number of Lys and Arg residues, which may result in novel post-translational modifications. In *Aspergillus nidulans* (Pezizomycotina, Eurotiomycetes) the H4E variant protein level is low in mycelia. However, the encoding gene is well expressed at 37°C under nitrogen starvation. H4E localizes to the nucleus and interacts with H3, but its absence or overexpression does not result in any detectable phenotype. Deletion of only one of the of the two canonical H4 genes results in a strikingly impaired growth phenotype, which indicates that H4E cannot replace this canonical histone. Thus, an H4 variant is present throughout a whole subphylum of the ascomycetes, but with hitherto no experimentally detectable function.

## 1. Introduction

In eukaryotes, chromosomal DNA is organized in nucleosomes, where 146 bp of DNA are wrapped around a histone octamer composed of two copies of each of histones H2A, H2B, H3 and H4 [1,2]. In the nucleosome, a heterotetramer of H3 and H4 binds two heterodimers of H2A-H2B [1]. Nucleosomes are the building blocks of chromatin, a dynamic structure that is involved in DNA-related cellular functions including replication, recombination, DNA repair and transcription [3]. The numerous proteins that participate in these processes compete with and/or displace nucleosomal histones. Several mechanisms control chromatin accessibility, such as post-translational modifications of core histone tails, ATP-dependent nucleosome remodelling complexes and the substitution of canonical histones by specialized histone variants [3–9], which show sequence differences from their canonical homologues. These variants confer specific properties to their cognate nucleosomes, impacting structure, stability and dynamics of changes that govern specific biological functions.

H4 is deemed to be the most conserved histone. It undergoes different post-translational modifications mainly at the N-terminal tail, many of which have been shown to play important regulatory roles in processes such as transcriptional regulation and DNA damage repair [10–13]. Only a few H4 variants have been identified so far in very different and phylogenetically distant species [14–18]. In fungi, usually two genes encoding almost identical canonical H4 histones are extant [19], one of which is typically transcribed divergently from the gene encoding H3. In both *Neurospora crassa* [20] and *Aspergilli* [21,22] another H4 variant is extant, with a different intron/exon structure from the canonical H4 histones. These proteins are recognized as H4 variants by the conservation of the core central domain while showing divergent N- and C-terminal extensions. In *N. crassa* inactivation of both canonical H4 encoding genes leads to inviability, which strongly suggests that the variant histone H4 cannot replace the functions of the canonical proteins [20].

In this work we carried out an extensive genome screening of publicly accessible DNA databases searching for ORFs encoding putative H4-like histone variants within the fungal kingdom. This scrutiny revealed the presence of such histone variants in the ascomycetes throughout the subphylum Pezizomycotina, and in basal species of the subphylum Taphrinomycotina but also, surprisingly, in the phylum Glomeromycota. The cognate gene shows specific, well-conserved intron/exon organizations that are substantially different from those of the two canonical H4 genes. We show that in *Aspergillus nidulans* (Pezizomycotina, Eurotiomycetes) this gene, which we named *hheA* (ANID_12228*,* encoding the H4E protein, the ‘E’ for ‘enigmatic’), is well expressed in mycelia under specific conditions (under nitrogen starvation, at 37°C). Deletion or overexpression of *hheA* do not lead to any observable phenotype. We show that H4E localizes to the nucleus and interacts with H3, thus is most possibly included in at least some nucleosomes, where its function remains undetermined.

## 2. Results

### 2.1. Distribution, conservation and structure of H4E encoding genes in the fungal kingdom

We conducted a thorough search of possible H4E encoding genes in available databases. A search by tBLASTn using the peptidic sequence of the *A. nidulans* canonical H4.1 (AN0734) as *in silico* probe revealed H4E-encoding genes in most species of the Pezizomycotina, with some obvious episodes of loss, such as in the genus *Penicillium* (Eurotiomycetes). In the *Aspergilli* (Eurotiomycetes), *hheA* encoding H4E lies between *nudA* (encoding a dynein heavy chain) and the orthologue of the *svf1* gene of *S. cerevisiæ*, the synteny being maintained in the Eurotiomycetes and Dothideomycetes fungi, including the *Penicillia* (Eurotiomycetes), which lack the *hheA* gene. An *hheA* gene is absent in the orders of the Ophiostomatales (Sordariomycetes) and the Botrosphaeriales (Dothideomycetes), even if it is present in other orders of the same classes. Genes encoding H4Es are found also in early diverging species of Taphrinomycotina (*Taphrina* sp*.*, *Saitoella complicata* and *Neolecta irregularis*) but not in the crown species of that subphylum and are also found in some Glomeromycota (see electronic supplementary material, figure S1). No putative *hheA* sequences were found in the Saccharomycotina.

**Figure 1.**
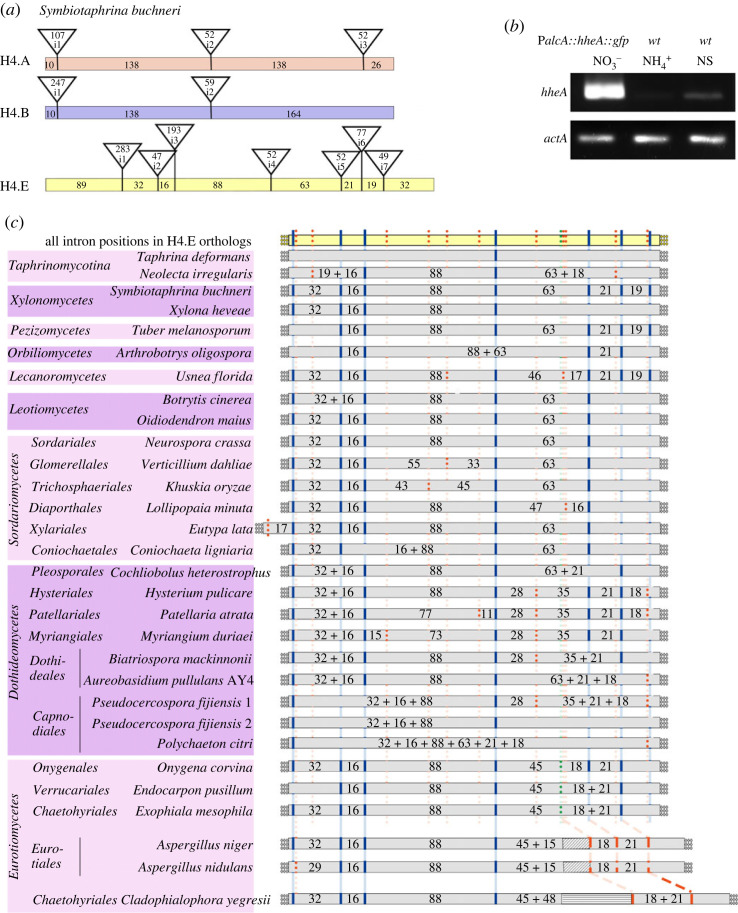
Overview of intron positions in H4.E genes in representative Ascomycota and RT-PCR assays to determine intron/exon organization in *A. nidulans*. (*a*) Comparison of the characteristic model intron/exon structure of the gene encoding H4E, found in *Symbiotaphrina buchneri*, with the intron/exon structure of the canonical H4 histones (H4.A and its paralogue H4.B). Coloured boxes refer to H4.A, H4.B and H4.E genes; vertical lines indicate intron positions in the coding sequence; texts within the triangles at the top of the vertical lines show the size in nucleotides of the cognate intron, and numbering of the intron (i.e. i1, i2, etc.). Numbers within the coloured boxes show the length of intron-separated exons. The typical gene model includes (from 5′ to 3′) a phase 2-intron, i1, separated from phase 1-intron i2 by exon 2 (29–32 nt), which in turn is separated by exon 3 (16 nt) from phase 2-intron i3, followed by an eighty 88 nt-exon 4. Phase 0-introns i4, i5, i6, and i7 are separated by exons of 60, 18, and 21 nt respectively. (*b*) RT-PCR assays carried out in *A. nidulans* to confirm the proposed intron/exon structure of *hheA*. mRNA samples were obtained from a *wt* strain grown at 25°C for 16 h on MM with 10 mM sodium nitrate (NO_3_^−^) as N-source, followed by shifting the mycelia for 4 h either to MM with 5 mM ammonium tartrate as sole N-source (${\mathrm{NH}}_{4}^{+}$) or with no N-source to induce nitrogen starvation (NS), and from a P*_alcA_*:*hheA*:*gfp* strain grown on 5 mM sodium nitrate and 0.1% fructose as sole carbon source for 16 h and inducing the P*_alcA_* promoter expression by adding 50 mM ethyl methyl ketone (EMK) and further incubating for 5 h. ɣ-actin (*actA*) was used as control. RT-PCR products were sequenced to confirm the proposed intron/exon structure (Genbank accession MW026189). (*c*) Schematic presentation of conserved intron positions in genes of H4.E orthologues from representative Ascomycota. A summary of intron positions occurring in Ascomycota are shown at the top of the draw, while species-specific intron positions are listed below. Blue vertical lines denote canonical intron positions, while dotted red lines denote non-canonical intron positions. Numbers in the boxes indicate the extent of coding region between two intron positions.

This patchy distribution could result from independent duplication events and/or be the result of loss after a duplication event. These two mechanisms could be extant independently in different nodes of the phylogeny. The intron/exon structure of all H4E encoding genes is completely different from that of canonical H4s. By comparative genomics we previously determined the common conserved gene model of seven species of *Aspergillus* to contain seven introns in conserved positions [21] (illustrated and detailed in figure 1*a*). The manually deduced intron/exon structure of the *A. nidulans hheA* gene (AN12228) (incorrect in the databases) has been experimentally confirmed in this work (Genbank accession MW026189) (figure 1*b* and electronic supplementary material, figure S2; see details in Materials and methods).

The specific H4E intron/exon structure suggests that the duplicated genes arose via cDNA, followed by neo-intronization, rather than from a genomic H4 copy. The predicted intron/exon structure of H4E encoding genes is strikingly conserved in the Pezizomycotina, e.g. it is identical in *Symbiotaphrina buchneri* (Xylonomycetes) [23] and in *Tuber melanosporum* (Pezizomycetes), with possible episodes of intron gain (such as specifically in the Dothideomycetes) and loss in different taxa. The seven intron/eight exon structure of the gene in *Symbiotaphina buchneri* was chosen as the typical gene model (figure 1*a*). It differs from the *Aspergillus* model in the position of two of the introns. In earlier divergent classes of the subphylum and in the Lecanoromycetes, an additional phase-1 intron is present 19 nt downstream of the most 3′ phase-0 intron in Eurotiomycetes, Leotiomycetes and Sordariomycetes. In Dothideomycetes (except in the Botryosphaeriales, which all lack the H4E gene), the intermittent exon is 18 nt long. By contrast, the fifth intron in the *Aspergillus* model only occurs in Eurotiomycetes. Usually, but not always, additional codons are present directly upstream of this class-specific intron; in the case of most Eurotiales and Onygenales, there are five additional codons, but certain taxa of Chaetothyriales include up to 16 additional codons in this location. Not considering the additional codons, the class-specific intron separates exons of 45 and 18 nt (in a few species of Eurotiomycetes), and these latter replace one exon of 63 nt bounded by position-conserved phase-0 introns in other taxonomical classes of Pezizomycotina. The additional codons in this location within the histone core region are unique to the Eurotiomycetes (figure 1*c*).

The intron/exon structure of the basal species of the Taphrinomycotina shows some intron positions in common with those in the Pezizomycotina (figure 1*c*), which would be consistent with a monophyletic origin for the H4E encoding gene in these two taxa. This intron/exon conservation does not extend to the Glomeromycota, where only one intron is extant in the H4E encoding genes. This intron, which is in phase 0, is one nucleotide downstream from intron i1 in figure 1*a*, which is in phase 2. This intron position has been experimentally confirmed in *Rhizophagus irregularis* (accession GW123471). Since intron sliding is a known phenomenon [24–26] we cannot distinguish this apparent near coincidental intron position as the result of the sliding of a cognate intron from that of an independent near-coincident intronization event.

**Figure 2.**
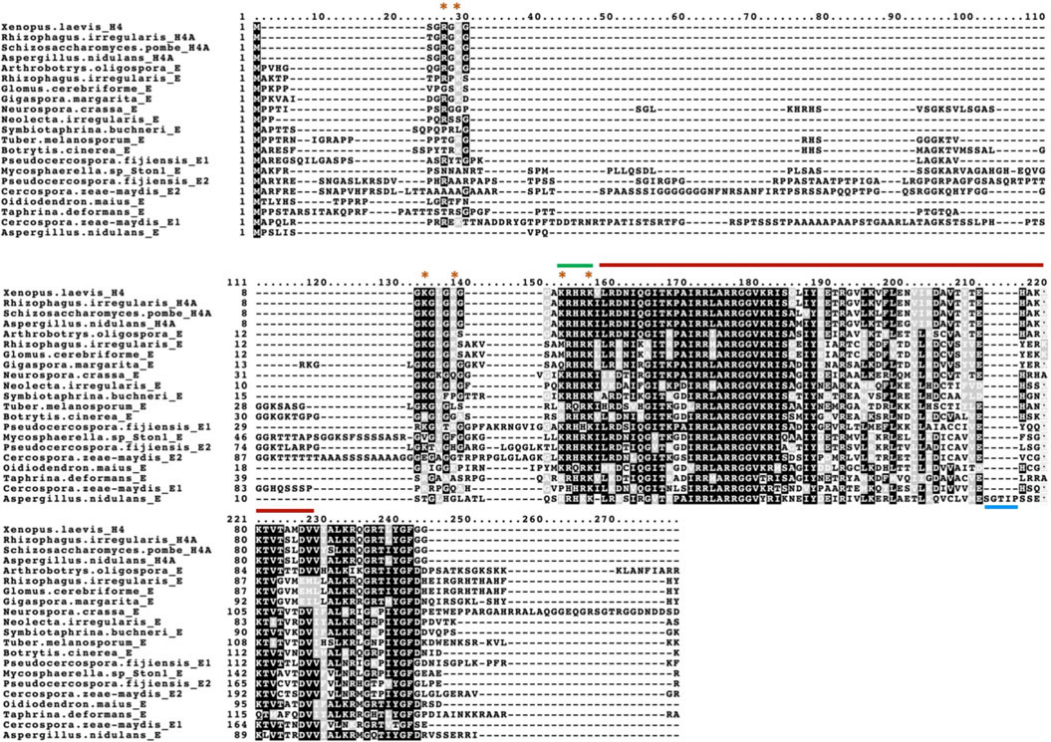
Variation of sequence of H4Es in representatives of different fungal clades. *A. oligospora* (Pezizomycotina, Orbiliomycetes); *N. crassa* (Pezizomycotina, Sordariomycetes); *S. buchneri* (Pezizomycotina Xylonomycetes); *T. melanosporum* (Pezizomycotina, Pezizomycetes); *B. cinerea* and *O. maius* (Pezizomycotina, Leotiomycetes); *A. nidulans* (Pezizomycotina, Eurotiomycetes); *R. irregularis*, *G. cerebriforme* and *G. margarita* (Glomeromycotina, Glomeromycetes); *P. fijiensis*, *C. zeae-maydis* and *Mycosphaerella* sp*.* Ston1 (Pezizomycotina, Dothideomycetes); *N. irregularis* (Taphrinomycotina, Neolectomycetes); *T. deformans* (Taphrinomycotina, Taphrinomycetes). Red bar: core histone-fold domain; green bar: N-terminal tail basic patch; blue bar: five amino acid insertion found exclusively in the *Aspergilli*; orange stars: residues reported as modification targets in canonical H4s. The canonical H4 histones of *Xenopus lævis*, *Aspergillus nidulans* and *Schizosaccahromyces pombe* are included as references to canonical H4 conservation. Alignments carried out with MAFFT L-INS-i with default settings and visualized with Boxshade.

**Figure 3.**
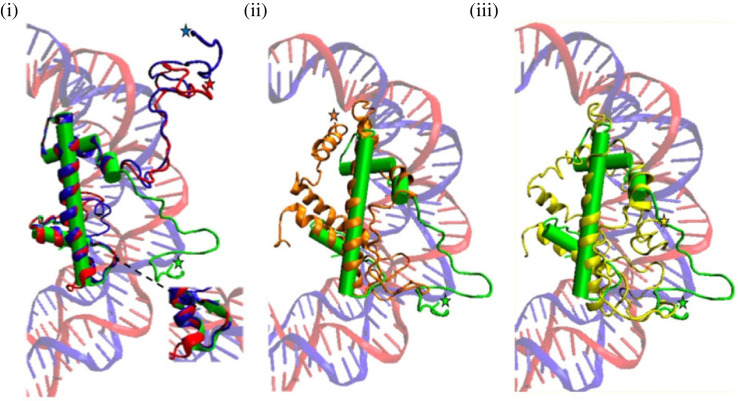
Predicted structures of selected H4Es from those aligned in figure 2. All structures were predicted with I-Tasser and drawn with VMD (see Materials and methods). The molecules were selected either for their putative independent evolutionary origin (see text) or for their striking sequence divergence. In each case the most probable model proposed by I-Tasser was chosen. (i) *A. nidulans* H4E (red) and *Rhizophagus irregularis* (Glomeromycota) H4E (blue) are superimposed to one of the *Xenopus laevis* canonical H4 molecules (solid green) in the nucleosome model 1kx5.pbd [32]. The inset to the right of the panel highlights a *β* turn present in the canonical histone but absent in both H4E models. (ii) H4E from *Mycosphaerella* sp*.* Ston1 (in orange) superimposed as below. (iii) H4E1 from *Cercospora zeae-maydis* (in yellow). Stars filled with cognate colours indicate the N-terminus of each protein.

The H4E deduced proteins present some specific characteristics as compared to canonical H4 histones (figure 2). Both the N- and C-terminal tails are longer in H4Es, and the N-terminus is especially divergent in terms of sequence. Noteworthy, in many of the analysed species, some (or even most) of the putative target residues for histone modifications present in canonical H4 N-terminal tails [27–29] are absent in H4E. The histone fold domain is quite conserved; interestingly, the Eurotiomycetes present a taxon-specific (but not ubiquitous) insertion of 5 to 16 amino acids (TIPSS sequence in *A. nidulans*) near the terminus of this domain. Even if not fully conserved, a basic patch [30,31] can be clearly identified at the end of the N-tail (figure 2 and electronic supplementary material, figure S3). The sequence divergence of histones H4E compared to canonical H4 possibly implies variations in the predicted structures of the proteins (figure 3). A complete list of deduced H4E sequences is included as electronic supplementary material.

### 2.2. Phylogeny of H4E encoding genes

It is reasonable to suppose that H4E encoding genes arose from one or several duplication event(s) of a canonical H4 encoding gene. To elucidate the origin of the H4E variant we constructed a number of maximum-likelihood phylogenetic trees incorporating canonical H4 genes from 497 fungal species and using H3 histone sequences as an outgroup. The phylogenetic analysis is complicated by the fact that histones are short proteins and thus the phylogenetic signal is rather weak. We could not obtain a stable phylogeny after trimming ambiguously aligned regions, drastically increasing taxon sampling and choosing different alignment algorithms (see Material and methods). We carried out a best ML tree search with ten random starting trees. If the dataset contained a strong phylogenetic signal, the resulting ten trees should have been topologically similar. In our case the ten tree searches ended with very distinct topologies even if the likelihood values were very close to each other (electronic supplementary material, figure S1).

While the H4E encoding genes seem monophyletic in the Pezizomycotina and in the Glomeromycotina due to the conservation of their intron/exon organization, the phylogenetic analysis does not resolve the origin of the H4E encoding genes in the basal Taphrynomycotina, which map in two distinct clades with separate and improbable phylogenetic positions. We note however that two trees (F and G) comfort the idea of an independent duplication origin of the H4E encoding genes in the Glomeromycota, in agreement with their intron/exon structure (see above). In spite of being extensive, the used dataset does not include sufficient information to result in a stable phylogeny.

While searching for H4E variants in different fungal species it come to our attention that in two clades that do not contain an H4E, the extant H4 proteins diverge from the canonical H4. These are the early divergent Microsporidia and Rozellidae, and some species of Trichomonasceae (Saccharomycetales). Specifically in the intracellular parasite *Rozella allomycis* five H4 paralogues, all showing different degrees of divergence from the conserved H4 sequence, are extant. This is illustrated in electronic supplementary material, figure S4.

### 2.3. The deletion of *hheA* in *A. nidulans* does not lead to any obvious phenotype

To assess the function of H4E, an *A. nidulans hheA* knock-out strain was obtained by replacing the complete coding sequence (CDS) with the *A. fumigatus riboB* (*riboB_Af_*) selection marker gene. Transformants bearing single integration events could be selected as described in Materials and methods. Thus, *hheA* is nonessential. When comparing the phenotypes of *wt* and *hheAΔ* strains grown on different carbon and nitrogen sources, at 25°C and 37°C, no differences were evident (electronic supplementary material, figure S5). Both strains conidiate normally and show the same rate of conidia viability (approx. 97%). Histone H4E could play a role in protecting DNA from damage and thus we assayed the sensitivity of *hheAΔ* strains to H_2_O_2_ and UV. Survival curves of wild-type and H4E-deleted strains are indistinguishable (electronic supplementary material, figure S6). We also checked for DMSO (dimethyl sulfoxide) sensitivity of *hheAΔ* strains. Following a proposal of Bartel & Varshavsky [33], extended by Goldman & Morris [34], if a protein becomes essential in a *hheAΔ* context and this protein happens to be sensitive to cell-penetrating solvents, strains lacking *hheA* could be DMSO-sensitive. Again, no differences between wild-type and mutant strains could be observed (electronic supplementary material, figure S7); however, it should be noted that our experiments only address an effect on overall cell viability and would not detect differences in damage at specific DNA sequences.

Secondary metabolism has been shown to be regulated by chromatin structure and histone post-translational modifications in *A. nidulans* [35,36]. To check a possible involvement of histone H4E in the regulation of secondary metabolite production, we monitored sterigmatocystin (STC) production in *hheA* and *hheAΔ* strains using thin-layer chromatography. The *veA1* mutation, present in most laboratory strains, alters the STC producing capacity of *A. nidulans* [37,38]. We thus deleted *hheA* in both *veA1* and *veA^+^* backgrounds with no obvious differences in STC production (electronic supplementary material, figure S8). The *hheA* deletion does not affect the sexual cycle when checked by selfing or outcrossing in either *veA^+^* or *veA1* backgrounds.

### 2.4. In *A. nidulans hheA* shows an increase in expression under nitrogen starvation conditions

**Figure 4.**
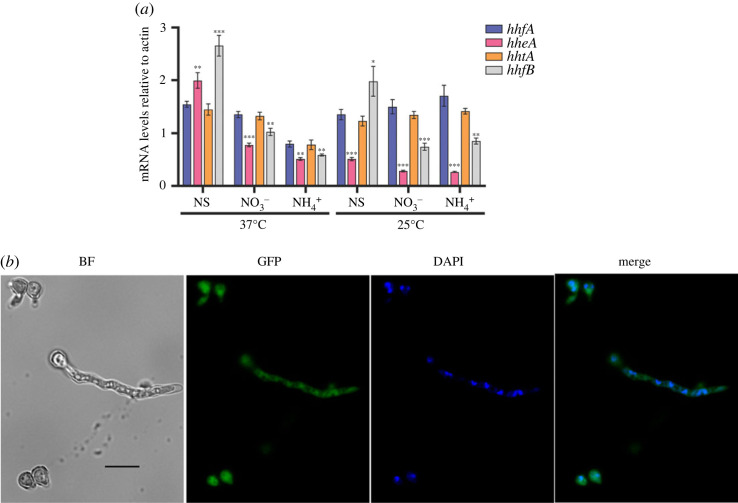
mRNA levels of the canonical H4.1 gene (*hhfA*), the enigmatic variant of the H4.1 histone gene encoding the H4E variant (*hheA*), the H3 histone gene sharing the promoter region with H4.1 (*hhtA*) and the canonical H4.2 histone gene (*hhfB*) measured by RT-qPCR in a *veA1* control strain (HZS.145). (*a*) Results, obtained by calculations according to the standard curve method [40], were normalized to ɣ-actin reference gene, *actA*. Standard deviations of three biological replicates are shown. The asterisks above the columns indicate the significance of the differences compared to the results on *hhfA* relative expression level. Significant differences were determined by using the Student's *t*-test. */**/*** indicates *p* < 0.05/0.01/0.001. Mycelia were grown on 10 mM sodium nitrate as the sole nitrogen source for 10 h at 37°C or 16 h at 25°C followed by transferring the mycelia to N-source-free medium (nitrogen starvation, NS), to 10 mM sodium nitrate (NO_3_^−^) or to 5 mM ammonium tartrate (${\mathrm{NH}}_{4}^{+}$) and further incubated for 2 h at 37°C or 4 h for 25°C. (*b*) Visualization of a H4E-GFP fusion driven by its own physiological promoter at 37°C under conditions of nitrogen starvation. Black scale bars, 10 μm. BF: bright-field microscopy; DAPI: nuclei staining; GFP: GFP signal.

We performed RT-qPCR on a wild-type strain to assess the expression of *hheA*, compared with that of H4 histone-coding genes *hhfA* (AN0734, encoding H4.1) and *hhfB* (AN2426, encoding H4.2), and the H3-coding gene *hhtA*, which is adjacent to *hhfA.* These two latter genes transcribe divergently, and probably share the same promoter region. Gene expression level was monitored on 5 mM ammonium tartrate, or 10 mM sodium nitrate as nitrogen sources, or under nitrogen starvation conditions following data from previous transcriptomic studies [39], at both 37°C and 25°C (figure 4*a*). At 25°C *hheA* is significantly downregulated compared to *hhfA* when grown under nitrogen starvation or on sodium nitrate or ammonium tartrate as sole N-sources, with fold-change (FC) values of 2.6, 5.3 and 6.5 respectively. At 37°C *hheA* was significantly downregulated on sodium nitrate and ammonium tartrate by 1.7 and 1.6 FC compared to *hhfA* but showed a positive fold change of 1.3 with respect to *hhfA* under nitrogen starvation. As expected, the expression levels of *hhtA* correlated well in all conditions with that of *hhfA* (figure 4*a*). Finally, at both assayed temperatures, *hhfB* showed significant lower expression on sodium nitrate and ammonium tartrate compared to *hhfA*, while it was upregulated under nitrogen starvation.

We also constructed strains with *hheA::(GA)_5_::gfp* and *gfp::(GA)_5_::hheA* fusions integrated homologously at the *hheA locus*, and thus driven by the physiological *hheA* promoter. We could not detect any fluorescence in hyphae grown on ammonium tartrate or sodium nitrate neither at 25°C nor at 37°C, or under nitrogen starvation at 25°C (electronic supplementary material S9). At 37°C some slight fluorescence under nitrogen starvation conditions could be observed in the nuclei, in line with RT-qPCR experiments (figure 4*b*). No fluorescence is detectable in conidiophores or ascospores from self-fertilization or outcrossing (electronic supplementary material, figure S10).

### 2.5. Overexpressed H4E-GFP localizes to the nucleus and does not produce distinctive phenotypes

**Figure 5.**
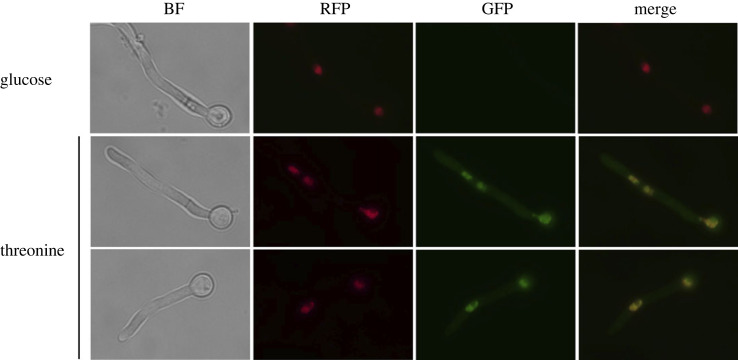
HheA::GFP colocalizes to the nucleus with a HhoA::mRFP fusion. Epifluorescence microscopy of a strain carrying a P*_alcA_*::*hheA::gfp* fusion when P*_alcA_* is repressed with 10 mM glucose (upper row), so no signal is seen in the GFP column, while green fluorescence is observed after P*_alcA_* induction with 10 mM threonine (second and third rows). Second column panels show the localization to the nuclei of histone H1 (HhoA) tagged with mRFP. Colocalization of GFP and RFP signals can be observed in the merge column. BF: bright-field microscopy. Growth conditions are in Materials and methods. A 100× oil immersion objective lens was used for visualization. Black scale bars, 10 µm.

We constructed plasmids carrying N- and C-terminally GFP-tagged H4E fusions regulated by the ethanol/threonine inducible promoter of *alcA* (P*_alcA_*) [41] and obtained strains bearing these integrated plasmids together with fusions of histone H1 (HhoA) with mRFP (monomeric Red Fluorescent Protein) as a nuclear marker (HhoA-mRFP) [42]. For both the C-tagged H4E (figure 5) and the N-tagged version (not shown), GFP fluorescence can be detected exclusively in nuclei and it shows significant overlap with histone HhoA-mRFP signals, which suggests a chromosomal localization.

We investigated the effect of H4E overexpression on the growth on different carbon and nitrogen sources (electronic supplementary material, figure S5), on the survival to UV exposure (electronic supplementary material, figure S6) and on the production of STC (electronic supplementary material, figure S8). No obvious phenotypes could be detected after any of these treatments.

### 2.6. Is H4E in the nucleosome?

**Figure 6.**
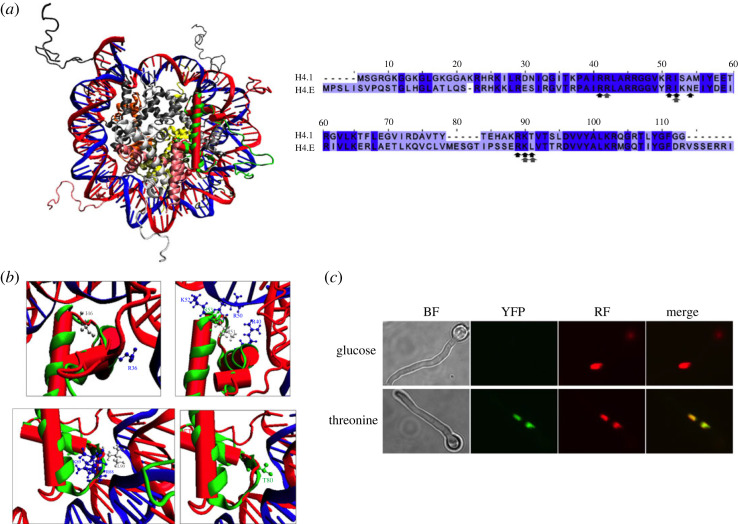
H4E fits in a nucleosome. (*a*) Left: superimposition of a model of H4E obtained through I-Tasser (https://zhanggroup.org/I-TASSER/) (solid red) with a nucleosome containing a canonical H4 derived from 1KX5 [43]. The second canonical H4 molecule is shown in orange; H2A: yellow and greyish white; H2B: tan and pink; H3: dark grey and intermediate grey. Right: alignment of the amino acidic sequences of *A. nidulans* H4.1 and H4E. Residues that contact DNA in H4E I-Tasser model are indicated with black arrows (Arg40, Arg50, Ile51, Lys52, Asn53, Arg88, Lys89 and Leu90), while those of the canonical histone are indicated with grey arrows (Arg36, Ile 46, Lys79, Thr80). (*b*) Detailed view of proposed DNA-H4E contact zones. Basic residues are coloured in blue, polar residues in green, and neutral in grey. (*c*) Bimolecular fluorescence complementation (BiFC) microscopy to show the interaction of H4E with canonical histone H3. H3::YFP_N_ and H4E::YFP_C_ fusions are overexpressed from the P*_alcA_* promoter when induced with threonine (lower panels). Glucose inhibits the expression of the fusions (upper panels). BiFC YFP signals are visible in green in the nuclei, where they co-localize with mRFP-tagged histone H1 (HhoA), seen in red. BF: bright-field microscopy. Merge: co-localization of YFP and mRFP signals. Growth conditions are detailed in Materials and methods. Black scale bars, 10 µm.

Modelling with I-Tasser [43] predicts a number of contacts of *A. nidulans* H4E with DNA (figure 6*a*,*b*). The interaction of H4E with H3 was shown experimentally by using the split-YFP version of the bimolecular fluorescence complementation (BiFC) assay [44]. We constructed plasmids for the expression of the N-terminal part of YFP (YFP_N_) fusion to histone H3 (H3::YFP_N_) and for the C-terminal part of YFP (YFP_C_) fusion to histone H4E (H4E::YFP_C_). Both gene fusions were expressed from the P*_alcA_* promoter. Co-expression of both plasmids in the presence of threonine leads to the appearance of fluorescence in the nuclei, co-localizing with a HhoA::mRFP fusion (figure 6*c*). When the co-transformed strain was grown on glucose, which is a drastic repressor of the *alcA* promoter [45], no YFP fluorescence was observed.

### 2.7. H4E is unable to compensate for the absence of canonical H4.1

**Figure 7.**
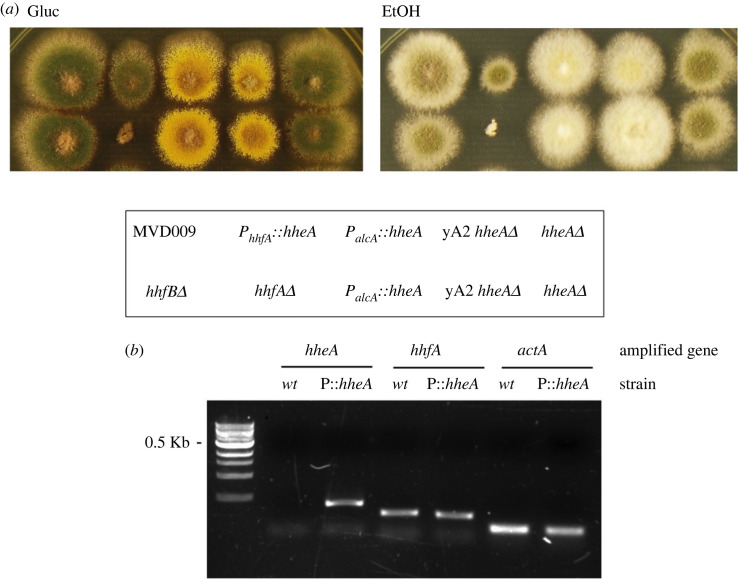
H4E is unable to compensate for the absence of canonical H4.1. (*a*) Colony growth of the strains indicated in the text box below, for 48 h at 37°C on the indicated media. MVD009 is the recipient strain for the P*_hhfA_*::*hheA* construction, where *hheA* is expressed under the control of the promoter of the canonical histone H4.1 (*hhfA*, AN0734); *yA2 hheAΔ* is the recipient strain for the P*_alcA_*::*hheA* construction; *hhfAΔ* and *hhfBΔ* bear deletions of the canonical H4 histones H4.1 and H4.2, respectively. (*b*) RT-PCR to compare the expression of *hheA* and *hhfA*, in a *wt* strain and in a strain expressing *hheA* under the control of P*_hhfA_* (P::*hheA*). *actA* was amplified and used as normalization control.

To obtain a strain carrying a deletion of the CDS of the canonical histone H4.1 (AN0734, *hhfA*), we replaced its complete CDS with the *A. fumigatus riboB* (*riboB_Af_*) marker. Deleted strains showed a strongly impaired growth, which suggests that this histone is necessary for normal physiological growth and neither its paralogue, the canonical histone H4.2 (AN2426, *hhfB*), nor the variant H4.E can complement H4.1 functions in a *hhfAΔ* strain. In line with this, deletion of the CDS of H4.2 has no apparent functional consequences (figure 7*a*).

We then assessed whether histone H4E can functionally complement the absence of H4.1 when expressed from the *hhfA* promoter. Hence, we substituted the endogenous *hheA* promoter with the *hhfA* gene promoter, and verified by RT-PCR that the resulting *hheA* expression levels were similar to those of *hhfA* in wild-type cells (figure 7*b*). The resulting strain has a mildly altered phenotype, which is more evident on ethanol than on glucose as carbon source (figure 7*a*). In this P*_hhfA_*::*hheA* context, the attempts to obtain *hhfAΔ* strains were unsuccessful, which suggests that H4E is not able to functionally substitute the main canonical H4 histone in *A. nidulans* nucleosomes.

## 3. Discussion

In this study we provide evidence of the presence of a novel H4 variant, H4E, in the subphylum Pezizomycotina of the ascomycetes and in basal species of the subphylum Taphrinomycotina, as well as in the Glomeromycota. While it is reasonable to suggest that genes encoding H4E arose from one or more than one duplication of a canonical H4 gene, and while a monophyletic origin of the H4E genes present in the Pezizomycotina and Taphrinomycotina is consistent with the extant intron/exon organization, no clear phylogenetic history could be deduced from the maximum-likelihood trees.

Among the fungi where an H4E encoding gene is present, *A. nidulans* and *N. crassa* have been used as model organisms to study a number of problems, including those relevant to chromatin structure [46–52]. *Aspergillus nidulans* is currently used in both the laboratories of Montevideo and Szeged and thus we have chosen it to verify the deduced intron/exon structure and for an experimental approach to the functional characterization of this novel histone variant. While the function of H4E may vary among the wide variety of the Pezizomycotina the few basal species of Taprhinomycotina where it is present and Glomeromycota which are mycorryhizal, our results may be relevant to at least the *Aspergilli*, including pathogenic species such as *A. fumigatus*, and more broadly to the Eurotiomycetes. In *A. nidulans hheA* is poorly expressed at 25°C, while it is well expressed at 37°C under specific conditions, such as nitrogen starvation. We showed that H4E can interact with histone H3; therefore, we may well suppose that it could be present in at least some nucleosomes. This is supported by the observation that both the native promoter-driven H4E-GFP (under nitrogen starvation, at 37°C) and overexpressed H4E-GFP co-localize with DAPI and HhoA-mRFP in nuclei. We could not detect any distinct phenotype of the *hheA-*deleted or overexpressing strains. The physiological role of H4E thus remains enigmatic. It may have a very specialized function in the cell, which could not be detected under our growth conditions. Under physiological conditions, neither H4E nor H4.2 are able to functionally rescue the severely impaired phenotype resulting from the deletion of *hhfA*, encoding one of the canonical H4 histones, H4.1. The deletion of the H4.2 encoding gene, *hhfB* (the paralogue of *hhfA* unlinked to H3), does not generate an observable phenotype. These results are at variance with what was observed in *N. crassa*, where the single inactivation of either of the genes encoding the canonical H4 proteins does not result in an observable phenotype, the double mutant being inviable [20]. This also suggests that H4v, the *N. crassa* H4E orthologue, is not able to functionally replace either of the canonical H4 histones.

One interesting observation is that the expression of *hheA* under the control of P*_hhfA_* results in a modest growth phenotype, which is not observed when the gene is overexpressed under the control of P*_alcA_*. This could be due to the fact that when *hheA* is driven by the *hhfA* promoter, it is regulated in the cell cycle together with H2B and H3 [53], which may allow the incorporation of H4E into nucleosomes. When the inducible *alcA* promoter drives the *hheA*, the *hheA* transcription is not in synchrony with the other core histone genes. Interestingly, *hhfB* is not regulated synchronically with the other core histones [41].

The most outstanding differences of H4Es with canonical H4s are the variable sequence and usually longer lengths of the terminal tails. Lys and Arg residues of H4 histones are targets of post-translational, regulatory modifications, and it is striking that H4E terminal tails show a paucity of these residues. Specifically, in *A. nidulans* the H4E N-terminal tail is 5 amino acids longer and the C-terminus is 7 amino acids longer than the cognate domains in H4.1 and H4.2. In the N-terminus, while six Lys and three Arg residues are extant in the canonical H4, only two Lys and two Arg are present in H4E. Many of the Lys residues of the H4 N-terminal tail have been shown to be acetylated and to be involved in different important cellular functions, such as chromatin modulation, heterochromatin formation, DNA repair [54,55] and deposition of canonical histone variants [56,57]. Hence, the absence of these Lys residues in H4E may result in nucleosomes less prone to regulatory modifications. One important sequence element found in the N-terminal tail of canonical H4 histones is the basic patch including Lys, Arg and His, comprising residues 16–24 [30,31]. X-ray crystallography of the nucleosome has suggested that this basic patch tends to form an α-helix that fits the acidic patch (a charged surface formed by highly conserved residues Glu56, Glu61, Glu64, Asp90, Glu91, and Glu92 from H2A, and Glu105 and Glu113 from H2B) of a neighbouring nucleosome [58]. This basic patch has been reported to be involved in the regulation of chromatin structure. In particular, K16 acetylation has been experimentally shown to interfere with the formation of compact chromatin structures, and to promote nucleosome mobility [58–60]. Moreover, the basic patch may have a role in regulating the activity of chromatin modifying complexes, such as SAGA [61] or ISWI [62]. Most H4Es show a more or less conserved basic patch (rich in Lys, Arg and His), but it is interesting that in many H4Es (including that of *A. nidulans*) the residue corresponding to canonical K16 is changed to an Arg or even to a non-polar residue.

H4 variants are extant in other organisms, and in all cases these variants fulfil different and specialized functions. In *Trypanosoma brucei*, a histone H4 variant (H4V) sharing 80% sequence identity with the already divergent canonical H4 has been found to be enriched at the transcription termination sites of the characteristic polycistronic transcription units produced by RNA pol II in trypanosomatids [14]. Another variant was reported in the urochordate *Oikopleura dioica*. In this testis specific histone three residues are different from the canonical H4, interacting with the acidic patch of the H2A-H2B dimer [18]. More recently, a human H4 variant, H4G, was identified. H4G lacks the characteristic C-terminal tail region of canonical H4 histones and the remaining sequence shares 85% identity with the corresponding portion of the canonical H4. H4G localizes to the nucleolus and was shown to regulate rDNA transcription in breast cancer cells [15,63]. A viral histone H4 encoded in the genome of the endoparasitoid wasp *Cotesia plutellae* bracovirus (CpBV) has been found to play a significant role in the parasite interaction with the host, the diamondback moth *Plutella xylostella.* CpBV H4 shows high sequence similarity with the insect histone H4, thus it possibly originated by HGT from *C. plutella*; however, and similarly to fungal H4Es, it includes a 38-amino-acid-long N-terminal tail, with a high lysine content. In the parasitized moths CpBv-H4 was shown to associate with other core histones to form octamers and to affect gene expression, including that of development and immune-response genes [16,17,63].

We have not been able to obtain a robust phylogeny of fungal H4 histone variants, even if intron positions suggest a monophyletic origin, at least within the Ascomycota. Sequence identity in the core domain is consistent with H4E variant arising from an original duplication of a canonical H4 encoding gene leaving open whether an independent duplication occurs in the Glomeromycota. An independent origin of H4E histones, from ancestral histone sequences, such as proposed for the histones of Marseilleviridae [64] is not supported by either phylogeny or core sequence comparisons.

While variant H4 histones occur less frequently and have been less studied that those in other core histones, they have appeared patchily throughout evolution, and most probably fulfil different functions in response to varied environmental conditions. Their presence in different fungal taxa amenable to experimental analysis should throw some light on the evolution of their functionality.

## 4. Materials and methods

### 4.1. *In silico* methods

Publicly available databases were searched with the *A. nidulans* H4.1 (AN0734) sequence using tBLASTn, at near default settings, except that the threshold was set at 1000 to allow the identification of more distant homologues, i.e. small genes encoding about 100 amino acids with dense intron/exon structures and multiple small exons. Resulting nucleotide sequences were scanned manually for intron/exon organization. When ESTS or cDNA sequences were available these were used to check the deduced gene models. For details, see electronic supplementary material. The H4E histone was modelled with I-Tasser (see [43] and references therein). The model was visualized with VMD 1.4.9 [65] and the H4E modelled structure was superimposed to a canonical H4 histone in the nucleosome structure 1KX5 [32] using the Multiseq [66] plugin included in VMD 1.4.9.

### 4.2. Strains and culture conditions

*A. nidulans* strains used and constructed in this work are listed in electronic supplementary material, table S1. Standard complete and minimal media (MM) for *A. nidulans* were employed, and supplements were added, according to the strains’ auxotrophic markers, at the following final concentrations: *p*-aminobenzoic acid, 1 µg ml^−1^; pyridoxine HCl, 0.5 µg ml^−1^; riboflavin HCl, 2.5 µg ml^−1^; uridine and uracil, 50 µg ml^−1^ each. The induction of P*_alcA_* was done on solid media by using glucose-free MM, supplemented with 2% ethanol as carbon source. In liquid media, 2-butanone, a gratuitous inducer of the *alcA* promoter, was added to a concentration of 50 mM to glucose-free MM containing 0.1% fructose as carbon source.

### 4.3. Molecular techniques

*Aspergillus nidulans* transformation was done as has been described previously by Tilburn *et al*. [67]. DNA and RNA from *A. nidulans* were prepared as described previously by Specht *et al*. [68] and Lockington *et al*. [69]. Plasmid DNA from *E. coli* was prepared as described by Sambrook *et al.* [70]. All plasmids and constructions were sequenced (Macrogen Sequencing Services, Korea), to verify the absence of PCR-introduced mutations. Gene replacement cassettes were constructed by using Double-joint PCR [71], as further detailed in the following sections. The cassettes were then transformed in the appropriate strains. The effective integration to the corresponding target loci was checked by PCR.

### 4.4. Construction of gene fusions and mutant strains

To obtain a *hheA* knockout strain, the cassette contained the *Aspergillus fumigatus riboB* marker (*riboB_Af_*), flanked by the 5′- and 3′-*hheA* flanking regions, to direct the integration of the cassette to the locus. The cassette was introduced in strain MVD009 (*riboB2 pyrG89 pyroA4 nkuAΔ::argB veA1*) and transformants selected by their ability to grow in the absence of riboflavin.

For the C-terminal tagging of H4E with GFP, a cassette was constructed by fusing: (a) the *hheA* 5′-upstream and CDS regions; (b) a fragment containing (Gly-Ala)5-GFP plus *A. fumigatus pyrG* (*pyrG_Af_*) as selection marker; and (c) the *hheA* 3′-flanking sequence. For N-terminal GFP-tagging of H4E, the construction of the cassette implied the fusion of five fragments: (a) the *hheA* 5′-flanking sequence; (b) GFP (without STOP) + (Gly-Ala)5; (c) the *hheA* CDS and the 3′-flanking sequence; (d) *pyrG_Af_* as marker; and (e) the *hheA* 3′- flanking sequence. Both N- and C-terminal GFP-tagging cassettes were transformed into a *pyrG89 pyroA4 riboB2 nkuAΔ::argB ΔhheA::riboB_Af_ veA1* strain (MVD500); transformants were selected for their ability to grow in the absence of uridine and uracil.

The constructions to overexpress *gfp*-tagged versions of H4E, expressed under the control of the P*_alcA_*, were constructed by fusing the following fragments. For the P*_alcA_*::*hheA*::(GA)5::*gfp* cassette: (a) the P*_alcA_* promoter; (b) the *hheA* CDS, lacking the STOP codon; (c) (Gly-Ala)5-GFP; and (d) the T*_trpC_* terminator. To generate the P*_alcA_*::(GA)5:: *gfp*::*hheA* cassette: (a) a 409-bp fragment containing the P*_alcA_* promoter; (b) the GFP + (Gly-Ala)5 fragment; and (c) the *hheA* CDS and 3′- flanking sequence. The cassettes were cloned into plasmids bearing the *pabaA* gene as selection marker and introduced in a *yA2 hheAΔ*::*riboB_Af_ riboB2 pyrG89 pyroA4 pabaA1 veA1* strain (MVD503), and transformants able to grow in the absence of *p*-aminobenzoic acid were selected.

To express *hheA* under the control of the *hhfA* gene promoter, the constructed cassette was composed of: (a) the *hheA* 5′-flanking region; (b) the *riboB_Af_* marker; (c) the intergenic region between divergently expressed genes *hhfA* and h3; and (d) the *hheA* coding region. The resulting cassette was transformed into a *pyrG89 pyroA4 riboB2 nkuAΔ*::*argB veA1* strain (MVD009), and transformants were selected on MM plates without riboflavin. The integration to the *hheA* locus was assessed by PCR.

### 4.5. Epifluorescence microscopy

Samples for fluorescence microscopy were incubated directly on glass bottomed dishes (Cell E&G LLC, Houston, USA) at 25°C for 14–16 h protected from light, in liquid MM supplemented as described in §4.1, according to the strains’ auxotrophic markers. To induce expression of GFP-fusions driven by P*_alcA_*, medium was replaced with adequately supplemented, glucose-free MM with 10 mM threonine as sole nitrogen and carbon source. Epifluorescence microscopy was carried out using an Olympus inverted microscope CKX31 with U-MNIBA3 (for GFP) or U-MWIG3 (for RFP) filters, documented with a Hamamatsu Orca Er camera and processed using IMAGE PRO v. 6.0 software. Microscope facilities belonging to the Cellular Biology Platform, Institut Pasteur de Montevideo were used.

### 4.6. Sensitivity assays

UV sensitivity curves were determined as described by Käfer & Mayor [72]. The used UV dose was 1.6 J m^−2^ s^−1^ and times of exposure were 0, 15, 30, 45, 60 and 90 s. To assay DMSO sensitivity, conidiospore suspensions were prepared, and spores were counted in a Neubauer chamber. 150 conidia were plated on complete medium and on complete medium containing 0.1–0.5% DMSO and incubated at 25 or 37°C [34]. The number of colonies grown on individual plates were counted after 72 or 48 h respectively, and the morphology and radial growth of the colonies were monitored. Sensitivity to H_2_O_2_ was assayed on conidiospores after Han *et al.* [73] and Kawasaki *et al.* [74]. 1 ml of a conidiospore suspension containing approximately 10^5^ spores was incubated for 30 min at 20°C, with final concentrations of 0–500 mM H_2_O_2_. 100 μl of a 1/50 dilution of the treated conidia was plated on MM with appropriate supplements and incubated at 37°C, after which colonies were counted and normalized to the number of colonies obtained on H_2_O_2_-free plates.

### 4.7. Extraction and detection of sterigmatocystin by thin layer chromatography

STC was extracted and detected as described by Karacsony *et al.* [75] with slight modifications. Briefly, five agar blocks were excised from the centre of 7-day-old *A. nidulans* colonies with a cork borer of 10 mm diameter. STC was extracted from the agar blocks by using 10 ml chloroform. The extracts were concentrated to 1 ml final volume at 65°C. Samples were loaded on Kieselgel 60 (Merck) plates after being normalized for the protein content of the samples (measured by the Bradford reaction) and the chromatogram was developed in toluol : ethylacetate : formic acid (50 : 40 : 10 v/v/v). Secondary metabolites were detected and recorded under UV light (366 nm), after being submerged in 10% AlCl_3_ in ethanol, and heated to 100°C for 1 min. Identification of STC was carried out with a STC standard (Sigma).

### 4.8. Reverse transcriptase PCR assays

To verify the *hheA* intron/exon structure mRNA was extracted from a *hheA^+^* strain grown for 16 h at 25°C on 10 mM sodium nitrate as sole nitrogen source and then for an additional 4 h in the presence of ammonium tartrate (5 mM) or under nitrogen starvation conditions. According to published data *hheA* is expressed to low levels in these conditions [39]. Additionally, we purified mRNA from a strain carrying the P*_alcA_*::*gfp*::H4E construct. In this case, conidia were grown for 16 h at 25°C on 10 mM sodium nitrate as sole nitrogen source and 0.1% fructose as sole carbon source and then further cultured for 5 h after inducing the P*_alcA_*-driven expression by adding 50 mM ethyl methyl ketone (EMK) to the medium. Total RNA was DNaseI treated prior to setting the first-strand cDNA synthesis reaction, which was performed with Superscript III reverse transcriptase (Thermo Fisher Scientific) according to the supplier's manual, starting with 1.4 µg of total RNA and 200 ng of random hexamers (Thermo Fisher Scientific). Primers H4EG1N and GAGFP-H4EG1-R were used to specifically amplify 344 bp of *hheA*, with Hi Taq DNA polymerase (Bioron). The amplified fragments were sequenced and aligned to the genomic sequence of the gene, to confirm the presence and position of the seven deduced introns.

To assess the expression of *hheA* under the control of the P*_hhfA_*, a *hheA^+^* control strain and the P*_hhfA_*::*hheA* strain were grown for 16 h at 25°C on 5 mM ammonium tartrate as nitrogen source. cDNA synthesis and specific PCR amplification of the 344 bp *hheA*-specific fragment were performed as described above. 241 and 105 bp fragments of *hhfA* and *actA* (encoding γ-actin) respectively were amplified as controls, with the primers specified in electronic supplementary material, table S2.

### 4.9. RT-qPCR

Total RNA was isolated from mycelia grown on 37°C or 25°C, as detailed in the caption of figure 4 by using TRI Reagent (Zymo Research). RNA quality was assessed by using agarose gel electrophoresis, while DNA contamination of the RNA samples was checked by carrying out qPCR on 1 µg RNA samples with γ-actin coding gene (*actA*/AN6542) using actA ReTi F2-actA ReTi R2 primers. Samples showing higher than 32 cycle Cq values in the DNA contamination test were used for reverse transcription. cDNA synthesis was carried out with a mixture of oligo-dT and random primers using a RevertAid First Strand cDNA Synthesis kit (Fermentas). RT-qPCR was carried out in a CFX96 Real-Time PCR System (Bio-Rad) with Maxima SYBR Green/Fluorescein qPCR Master Mix (Fermentas) reaction mixture (94°C for 3 min followed by 40 cycles of 94°C for 15 s and 60°C for 1 min). Data processing was performed by the standard curve method [40]. Gene expression values of genes of interest were normalized to *actA*. The primers used are listed in electronic supplementary material, table S2.

### 4.10. Bimolecular fluorescence complementation analyses

For BiFC analyses, full-length *hhtA* (AN0733, encoding the canonical histone H3) and *hheA* coding sequences were amplified from genomic DNA, with primers bearing *Asc*I and *Pac*I restriction sites (see electronic supplementary material, table S2). Once digested, the H3-coding fragment was cloned in pDV7 [44] yielding plasmid pDV7-H3, bearing a P*_alcA_*::YFP_N_::*hhtA* fusion and the H4E-coding fragment in pDV8 [44], yielding plasmid pDV8-H4E, bearing a P*_alcA_*::YFP_C_::*hheA* fusion. In-phase fusion was verified by sequencing. Both plasmids were transformed into a *pabaA1 pyroA4 pyrG89 veA1* strain, and transformants were selected in the absence of uridine and uracil. To distinguish between specific and unspecific interactions, pDV7-H3 was co-transformed with pDV8 and pDV8-H4E with pDV7 as controls.

## Data Availability

*Aspergillus nidulans* histone H4 variant mRNA complete CDS was deposited in Genbank (Genbank accession MW026189). Nucleosome modelling was done based on structure 1KX5 X-ray structure of the nucleosome core particle, NCP147, at 1.9 Å resolution [32]. Supplementary material is available online [76].
